# Intrastromal Injections in the Management of Infectious Keratitis

**DOI:** 10.3390/pharmaceutics15041091

**Published:** 2023-03-29

**Authors:** Mihail Zemba, Madalina Radu, Sinziana Istrate, Otilia-Maria Dumitrescu, Mihai Alexandru Ionescu, Andrei Vatafu, Ileana Ramona Barac

**Affiliations:** 1 Department of Ophthalmology, University of Medicine and Pharmacy “Carol Davila”, 050474 Bucharest, Romania; 2 Department of Ophthalmology, “Dr. Carol Davila” Central Military Emergency University Hospital, 010825 Bucharest, Romania

**Keywords:** intrastromal injection, intracorneal injection, bacterial keratitis, fungal keratitis, viral keratitis, microbial keratitis, recalcitrant keratitis, targeted therapy

## Abstract

Infectious keratitis is a major global cause of vision loss and blindness. Prompt diagnosis and targeted antibiotic treatment are crucial for managing the condition. Topical antimicrobials are the most effective therapy for bacterial keratitis, but they can lead to unsatisfactory results due to ocular perforation, scarring, and melting. Intrastromal injection is a newer technique for delivering antimicrobials directly to the site of infection and has been successful in treating severe, treatment-resistant infectious keratitis, especially when surgery is not recommended. In cases where deep stromal disease is resistant to topical treatment, intrastromal antimicrobial injections may be necessary to achieve higher drug concentration at the infection site. However, the use of intrastromal antibiotics is limited, as topical antibacterial agents have better penetration than antifungal agents. Bacterial and fungal keratitis have been extensively researched for intrastromal medication injections, while there is limited evidence for viral keratitis. This review emphasizes the potential of intrastromal antimicrobial injections as an alternative for managing severe refractory infectious keratitis. The technique offers direct targeting of the infection site and faster resolution in some cases compared to topical therapy. However, further research is needed to determine the safest antimicrobials options, minimal effective doses, and concentrations for various pathogens. Intrastromal injections may serve as a non-surgical treatment option in high-risk cases, with benefits including direct drug delivery and reduced epithelial toxicity. Despite promising findings, more studies are required to confirm the safety and efficacy of this approach.

## 1. Introduction

Diseases that affect the cornea remain a major cause of monocular blindness worldwide. Corneal opacities, which are determined by a wide variety of infectious and inflammatory eye diseases, lead ultimately to functional blindness [1]. Infectious keratitis, also known as an infectious corneal ulcer, represents an infection of the cornea due to the proliferation of microorganisms associated with inflammation and corneal tissue destruction. Infectious keratitis can be caused by pathogens such as bacteria, fungi, viruses or parasites [2]. Clinically, infectious keratitis is established by the presence of corneal ulceration alone or associated with stromal infiltration, anterior chamber reaction or signs of conjunctival injection [3]. In any etiology of infectious keratitis, topical treatment remains the mainstay of treatment because of the ease of administration and patient compliance. However, the penetration of many topical antimicrobial drugs is not ideal to treat deep infectious keratitis, such as fungal keratitis, due to extremely limited ocular bioavailability [4]. To solve these issues, alternative approaches have been explored, including intracameral and intrastromal injections. Similar site-directed drug deposits, such as intravitreal injections and posterior sub-tenon injections, have been made in posterior segment diseases [5]. The main benefit of a targeted drug delivery system is that it makes certain that the right amount of medication is delivered to the infection site; as a result of bypassing the corneal esterase’s first-pass metabolic process, a higher dosage of the drug is delivered directly to the site of action. Furthermore, it can be a good alternative when poor adherence to the prescribed treatment is suspected [6].

Excisional keratectomy coupled with focal cryotherapy, penetrating keratoplasty, collagen cross-linking with photoactivated riboflavin, amniotic membrane inlay, conjunctival flap surgery, and tarsorrhaphy are a few other interventions that have been suggested to treat these cases of severe and persistent infectious keratitis [7]. These approaches have disadvantages of their own, but intrastromal injections of antimicrobials that are directly targeted at the ulcer site have been shown to be a successful alternative for bypassing these complex options.

## 2. Materials and Methods

A literature search was performed using an electronic database of the PubMed interface for relevant articles using the following key words or words combinations: “intrastromal injections”, “intracorneal injections”, “corneal ulcer”, “infectious keratitis”, “bacterial keratitis”, “fungal keratitis”, “viral keratitis”, “targeted therapy”. All relevant articles written in English were included in this review. Articles written in other languages were also included if there was an English language abstract with a comprehensive summary of the article. The references of each article were also analyzed to obtain further relevant articles. Prospective studies and randomized clinical trials were included in this article. However, case series and case reports were also included where considered necessary, given the lack of data on intrastromal injections in infectious keratitis. Studies on animal models and studies not relevant to the topic were excluded. We retained 60 published articles prior to December 2022.

## 3. Results

### 3.1. Bacterial Keratitis

Bacterial keratitis (BK) is the most prevalent reason for microbial keratitis [3,8]. The use of contact lens is the major risk factor for this pathology in developed countries, whereas trauma is the greatest risk factor in underdeveloped countries [5]. Apart from these, previous topical steroid use, ocular surface disease, ocular trauma, previous keratitis and different corneal diseases are considered predisposing risk factors for bacterial keratitis [3,9,10,11]. The gold-standard treatment at the moment is represented by empiric broad-spectrum topical antibiotics. The current guidelines recommend monotherapy with fluoroquinolones (ciprofloxacin 3 mg/mL, ofloxacin 3 mg/mL, moxifloxacin 3 mg/mL, besifloxacin 6 mg/mL, levofloxacin 15 mg/mL, gatifloxacin 3 mg/mL) [11]. A combination of cephalosporine or vancomycin and an aminoglycoside can be used as well [11].

In some cases, topical antibiotic therapy is exceeded in deep stromal disease. To address this issue, antibiotic intrastromal injections were utilized to enhance medication concentration at the area of infection. The use of intrastromal injection in bacterial keratitis is unfortunately not well documented in the literature. The experience with intrastromal antibiotic treatments for bacterial keratitis is minimal, despite the fact that intrastromal antifungal drugs have been utilized to treat refractory fungal keratitis. However, there are only a few cases of intrastromal antibiotic injections successfully treating bacterial keratitis. Two of these cases reported cases of infectious crystalline keratopathy (ICK) recalcitrant to topical therapy [12,13,14]. Infectious crystalline keratopathy represents a chronic corneal infection characterized by infiltration in a needle-like configuration in the anterior corneal stroma; therefore, this makes it difficult to identify causative pathogens from superficial corneal scrapings [2]. Although ICK more frequently affects corneal transplants, it can also affect previously healthy cornea, particularly in individuals receiving immunosuppressive medication [15]. *Streptococcus mitis*, a gram-positive bacterium, was indicated to be responsible for the most cases of this pathology, [2] but *Streptococcus pneumonia* [16], *Haemophilus aphrophilus* [17], *Pseudomonas aeruginosa*, *Candida* fungal species [18], gram-negative bacteria [19], *Acanthamoeba* [20], and *Diphtheroids* [21] were also causative organisms among published reports.

An important issue that is brought to light by multiple authors is that of biofilm formation being implicated in the pathogenesis of infectious crystalline keratopathy [12,13]. Corneal biofilms are defined as a functional group of microorganisms structured into an exopolymer matrix which interferes with human immune responses and shields the organisms from antibiotics. Biofilms present a substantial challenge to the effectiveness of antimicrobial agents, due to several factors that contribute to their reduced efficacy. First, the extracellular polymeric substance (EPS) matrix that surrounds the microbial cells in biofilms hinders the diffusion of antimicrobial compounds, resulting in impaired penetration and suboptimal drug concentrations at the target site [22]. Second, biofilm-associated microbial cells display diverse metabolic states, including dormant or slow-growing cells that are less susceptible to antimicrobial agents targeting active growth [23]. Third, genetic adaptation and resistance can emerge within biofilms, facilitating horizontal transfer of resistance genes among microbial cells and further compromising the effectiveness of antimicrobial agents [24]. Finally, the biofilm microenvironment can create localized pH and oxygen gradients, which impact the activity of antimicrobial agents, further reducing their effectiveness against the microorganisms [25]. As a result, because the organisms’ protective biofilm prevents antibiotics from reaching therapeutic levels at the location of the microbial cluster, topical antibiotics are inefficient in patients with ICK [26].

Khan et al. are first to report on the use of intrastromal injections with antibacterial agents [12]. They administered intrastromal cefuroxime 250 µL/mL to successfully treat a case of infectious crystalline keratopathy secondary to *Streptococcus paranguis*. To reveal the corneal stroma and biofilm behind the mucous plaque and epithelium, the patient first underwent debridement of those layers. The lesion and surrounding stroma were then injected intrastromally with one milliliter cefuroxime using a 30-gauge anterior chamber Rycroft 0.30 × 22 mm, using the “hydration technique” [12]. In this case, cefuroxime was chosen above other antibiotics, such vancomycin, not only for its sensitivity and low inhibitory concentration, but also because it is less harmful to the ocular surface [12]; although, incidents of accidental intrastromal injections of gentamicin (20 mg/0.5 mL) [27] and vancomycin (1 mg/0.1 mL) [28] without obvious harmful effects have been described.

In line with the previous study, Martinez-Velazquez et al. also reported a unique case of successful management of an immunocompromised patient with infectious crystalline keratopathy and ocular graft vs. host disease (GVHD) [13]. They used sequential intrastromal antibiotic injections. The first intrastromal injection was with 0.1 mL of 1 mg/0.1 mL cefuroxime associated with 0.1 mL of 0.5 mg/0.1 mL moxifloxacin intracamerally. This previous injection failed to control ICK, and five weeks later the second intrastromal injection was administered. The latter consisted of 0.1 mL of 0.5 mg/0.1 mL moxifloxacin and 0.06 mL of 1 mg/0.1 mL cefuroxime and was injected in a circular pattern surrounding the lesion. The main goals of the case and the approaches used to deliver the treatment were to avoid a Descemet’s membrane separation from the considerable stromal edema by delivering the least amount of antibiotics possible, and by positioning the needle tracks outside of the visual axis [13,29].

Liang et al. [27] reported another case of resistant bacterial keratitis. They used a 30-gauge needle to deliver a single intrastromal injection of 0.02 mL of tobramycin 0.3%. After six months, the keratitis became dormant, and five years later, there was no sign of a recurrence [27]. Pak et al. recently described for the first time in the literature a triple-bacterial keratitis caused by penicillin-resistant S. aureus, pan-sensitive S. epidermidis, and *Achromobactin* species with unknown antibiotic sensitivities treated successfully with intrastromal antibiotic injection [30]. When topical treatment failed to treat the keratitis, a new strategy was used and 0.2 mL of 0.5% moxifloxacin was administered intrastromally, precisely at the edge of the infiltrate. Five days after the first injection, there was some improvement, but unsatisfactory, so it was decided to administer the second injection with the same dose 2 weeks later, resulting in complete remission of the keratitis [30].

Despite the fact that there are currently no guidelines regarding antibiotic agent selection, concentrations, or injection volumes, the concentration used in cases described above was chosen in accordance with the recommendations used for intracameral injection in cataract surgeries across the globe [14].

### 3.2. Fungal Keratitis

Mycotic or fungal keratitis accounts for approximately 1–45% of the total burden of microbial keratitis [6,31]. While contact lens use has become a significant risk factor for fungal keratitis in developed countries, trauma with vegetable material or items contaminated with soil is the most frequent risk factor in developing countries [32,33]. The three most prevalent fungi that cause corneal pathology are *Aspergillus* spp., *Fusarium* spp., and *Candida* spp. [34]. Lalitha et al. [35] showed that infection with Aspergillus spp. is an important risk factor for treatment failure.

Due to its difficulty of treatment and poor prognosis, the management of recalcitrant or deep fungal keratitis required alternative routes besides topical or oral administration of antifungal drugs. Firstly, the intracameral route was used as a mode of drug delivery, which was soon followed by intrastromal injections of antimycotic drugs, placing these routes in the concept of targeted drug delivery.

An increasingly useful technique for treating corneal infections with a known or suspected fungal pathogen is an intrastromal injection of antimicrobial drugs. This is because fungal infections are more frequently deep stromal infections, and also because oral and topical antifungal medications usually have bigger molecular structures, they typically have suboptimal corneal penetration.

Deep mycotic keratitis (>50% of stromal depth) and resistant cases (not responding to standard treatment for at least two weeks) are indications for targeted therapy. All clinical studies and case reports analyzed the patients’ age, ulcer size, mean time between beginning of symptoms and hospital admission, and existence of and extent of hypopyon at the time of presentation. The primary outcomes were best spectacle-corrected visual acuity (BSCVA) after intervention, healing time, scar size, reduction in abscess size, resolution of epithelial defect, stromal infiltrates, and infection.

#### 3.2.1. General Management Guidelines

It is difficult to establish standardized recommendations for the management of fungal keratitis that are relevant to all settings because of variations in the facilities and treatment choices that are offered. Due to the decreased ocular penetration and efficiency of antifungal drugs, as well as the challenging identification of this infection to start an effective first treatment, fungal keratitis has poor clinical results. Antifungal medications, cycloplegics to relieve anterior uveitis, antibiotics for secondary bacterial injection if present, and surgical intervention are all used to treat fungal keratitis [36].

Natamycin (5% concentration) is the initial drug of choice for fungal keratitis. If worsening or no improvement is seen after two weeks of therapy, topical voriconazole 1% can be substituted or added in cases of *Fusarium* spp. and *Aspergillus* keratitis, and, in addition, topical amphotericin 0.15% can be substituted in cases of *Candida* spp. and *Aspergillus* spp. keratitis [33]. According to the TST (Topical, Systemic, and Targeted Therapy) Protocol [37], a systemic antifungal should be prescribed in cases of deep (>50% stromal depth) and large (>5 mm) ulcers. Oral medications such as voriconazole, ketoconazole, itraconazole, and fluconazole are the main systemic antifungal drugs; although, posaconazole, a new medication, has broad-spectrum activity against *Fusarium* spp., *Aspergillus* spp., and *Candida* spp. without any proven toxicity. If there is still a poor response after 7–10 days, intrastromal or intracameral injections with antifungal drugs are taken into consideration. Generally, these injections can be repeated up to four times, three days apart. If there is still a poor response despite targeted therapy, penetrating keratoplasty or other surgical alternatives are indicated [37].

#### 3.2.2. Antifungal Drugs

Based on their molecular makeup and mode of action, antifungal medications may be divided into several groups. These substances fall under the general categories of polyenes (amphotericin B, natamycin, nystatin), azoles (ketoconazole, miconazole, econazole, voriconazole, fluconazole, ravuconazole, itraconazole, posaconazole), pyrimidines, allylamines, echinocandins (capsofungin, micafungin, anidulafungin), and heterocyclic benzofurans. Polyenes and azoles compounds are often used as topical treatments for ocular mycotic infections. Natamycin 5% drops for filamentous fungi and amphotericin 0.5% for yeast-like fungus are the two most commonly used topical anti-fungal medications [38]. A new generation of azoles, voriconazole in particular, is being utilized more often because of its wide spectrum and superior ocular penetration profile. Azoles agents are employed as either adjunctive or alternative medicines in non-responding and recalcitrant fungal keratitis [39]. Regarding intrastromal injections, the most studied antifungal agents are voriconazole (VCZ), amphotericin B (AMB), and natamycin (NTM).

Various studies in the literature have reported conflicting results concerning targeted therapy with voriconazole (Table 1).

##### Voriconazole

The chemical family of azoles includes the antifungal medication voriconazole. Both parenteral and oral preparations are offered. Its topical ophthalmic usage is off-label and necessitates reconstituting the parenteral formulation to a solution with a concentration of 1% (10 mg/mL) before use [6,40].

Voriconazole is the most often used antifungal medication delivered intrastromally. Voriconazole targeted drug delivery has been examined for the treatment of fungal keratitis that has not responded to standard topical therapy. A significant drawback of topical antifungal treatment, limited drug absorption in cases of deep fungal corneal ulcers, is resolved by this mode of drug administration. It delivers a drug depot near the ulcerated site at a dosage of 50 µm/0.1 mL in five divided doses, from which the medication is progressively released into the affected tissue [33,41].

Two randomized controlled studies have compared intrastromal voriconazole injections with topical treatment alone [42,43]. A randomized controlled trial with 40 patients who had shown no improvement after receiving natamycin 5%, as a first line of treatment, was the first study published. Patients were assigned to receive either intrastromal injections of voriconazole 50 µg/0.1 mL or topical voriconazole 1% alone. A total of 19 out of 20 patients in the topical group and 16 out of 20 patients in the intrastromal group responded well to the therapy (*p* = 0.34). BSCVA, scar size, perforation rate, hypopyon resolution, and stromal infiltrate size were all taken into account by the authors as outcome factors. After therapy, the group receiving topical voriconazole had considerably higher visual acuity (*p* = 0.008). Additionally, the topical group’s ulcers healed 5.5 days sooner than the intrastromal group’s ulcers; although, there was no statistically significant difference between the two groups (*p* = 0.38) [42]. In the second clinical trial, called Mycotic Antimicrobial Localized Injection (MALIN), 70 patients with mycotic keratitis were treated with either natamycin 5% monotherapy or natamycin 5% plus intrastromal voriconazole. The authors came to the conclusion that the main treatment of moderate to severe filamentous fungal ulcers does not benefit from the adjuvant intrastromal voriconazole to topical natamycin 5% [43].

In spite of these results, some case studies have demonstrated that intrastromal voriconazole may be useful in treating deep infiltrates or stromal abscesses that have not responded to first- and second-line topical and systemic antifungal treatments (natamycin 5%, voriconazole 1%, oral itraconazole or ketoconazole) [37,41,44,45,46]. Prakash et al. reported the first series of three patients in which intrastromal voriconazole (50 µg/0.1 mL) was used in combination with topical natamycin 5% therapy to effectively cure deep-sealed persistent fungal keratitis [41]. Positive results were also documented in larger case series [37,44,45,46]. Patients with confirmed fungal keratitis who did not react to topical natamycin 5% or topical voriconazole 1% were given intrastromal voriconazole injections. Kalaiselvi et al. [46] observed a 72% treatment success rate in 18 of 25 eyes. Similarly, Konar et al. [45] reports a 70% success rate, 14 out of 20 patients. To attain an optimal response, the need for repeat injections was reported in 15% [46], 75% [45], and 80% [44] of patients, respectively.

Intrastromal VCZ injections have been used successfully for the treatment of secondary lamellar interface infection for late-onset infectious keratitis following Descemet stripping automated endothelial keratoplasty [47], recalcitrant *Acremonium* keratitis [48], *Alternaria* keratitis [49], post photorefractive keratectomy mycotic keratitis [50], and fungal infection of the phacoemulsification site tunnel [51].

##### Amphotericin B

The first medication to clinically treat mycotic keratitis was amphotericin B. AMB is a broad-spectrum antifungal polyene macrolide made by *Actinomyces Streptomyces nodusus*. AMB has fungicidal properties and, by its interaction with the ergosterol in fungal cell membranes, promotes cell death through hole creation and fatal alterations in membrane permeability. Its spectrum of action primarily targets *Candida* spp., *Aspergillus* spp., and *Cryptococcus*, while it is less effective against *Fusarium* spp. Topical administration with the standard method with a concentration of 0.15% (1.5 mg/mL) to 0.5% (5 mg/mL) solution results in poor ocular penetration, as well as cytotoxicity at high doses that causes punctate corneal erosions, epithelial defects, stromal oedema, and iritis, and requires access to a compounding pharmacy for manufacturing of the necessary dosage [38].

In the literature, it was emphasized that for improved treatment results, intracameral, intrastromal, and intravitreal amphotericin B should be used in conjunction with either topical therapy or in combination with other conventional medications. In a retrospective case series, Nada et al. [52] assessed the effectiveness of topical AMB (n = 27) and combination intrastromal injection of AMB (0.02 mg/mL) and topical fluconazole 2% (n = 41). A greater resolution rate (82.9% vs. 59.3%) and quicker healing (24 ± 6.4 vs. 39.6 ± 13.6 days) were both seen in the intrastromal group. With a high failure rate within the *Fusarium* subgroup, *Candida* species were detected in the majority of cases in this study.

Aydin et al. [53] evaluated the clinical effects of combining intrastromal voriconazole (0.05 mg/0.1 mL) and intrastromal amphotericin B (0.01 mg/0.1 mL) for the treatment of persistent fungal keratitis in 32 eyes that had previously been treated with a combination of topical voriconazole (1 mg/0.1 mL) and topical amphotericin B drops (0.15 mg/0.1 mL) for at least two weeks. In addition to topical treatment, 28 (87.5%) patients received a combination of intrastromal amphotericin B and intrastromal voriconazole therapy, which completely resolved their chronic fungal keratitis. The combination intrastromal injections have to be given once more after three days in order for the fungal keratitis to heal as expected. Four patients needed therapeutic penetrating keratoplasty due to the continuation of fungal keratitis in two of them and the advancement of keratitis, despite improvements in the mean best-corrected visual acuity values in the entire study.

There have been successful reports of treating severe resistant fungal keratitis by combining intrastromal AMB injection with another delivery method, such as intracameral or intravitreal injections. The first was that of a challenging case of recurrent fungal keratitis with endophthalmitis after a contaminated penetrating keratoplasty, in which amphotericin B was administered intrastromally into the cornea and intravitreally into the eye [54]. The corneal fungal plaques and the intraocular infection were both successfully treated with this therapeutic approach. In another study, nine eyes with severe fungal keratitis that were resistant to standard medical antifungal therapies and required possible surgical intervention were treated with a combination of intrastromal and intracameral injections of amphotericin B [55].

##### Natamycin

This polyene macrolide, which was first developed in the 1960s, has withstood the test of time and is the most scientifically supported treatment now offered for the treatment of filamentous fungal keratitis. *Streptomyces natalensis*, a bacterium, naturally produces it. A suspension formula with a 5% concentration (50 mg/mL) was developed. *Fusarium*, *Aspergillus*, *Alternaria*, *Candida*, *Cephalosporium*, *Colletotrichum*, *Curvularia*, *Lasiodiplodia*, *Scedosporium*, *Trichophyton*, and *Penicillium* are only a few of the mycopathogens that it is effective against. Natamycin’s weak ocular penetration has been one of its main drawbacks, leading physicians to search for medications with better bioavailability at the infection site to hasten the keratitis symptoms’ remission [38,56].

Even though voriconazole is thought to be superior, natamycin treatment was shown to provide better results in several of these investigations. The largest of these, the MUTT trial [57], a double-masked multicentric randomized control trial (n = 323) comparing topical natamycin (5% concentration) with voriconazole (1% concentration), revealed that natamycin was linked to better clinical and microbiological outcomes in smear-positive filamentous fungal keratitis, particularly in cases of *Fusarium* keratitis. When compared to the natamycin cohort, the voriconazole group had a higher risk of corneal perforation and a greater requirement for penetrating keratoplasty [57].

Due to its intrinsic properties, such as limited water solubility and ocular bioavailability, intrastromal usage of natamycin is less researched and less often employed. Recent three-way randomized controlled clinical research examined intrastromal amphotericin B (5 µm/0.1 mL), intrastromal voriconazole (50 µm/0.1 mL), and intrastromal natamycin (10 µm/0.1 mL) injections in fungal keratitis patients who did not respond to two weeks of topical natamycin 5% treatment (sixty eyes in total, with twenty in each group) [58]. Patients who were included had ulcers that were more than 2 mm in size and more than 50% stromal involvement. According to this study, the intrastromal natamycin group healed on average more quickly than the other two groups (*p* = 0.02). In terms of healing effectiveness, there was no indication of any difference between the groups (95% in the intrastromal voriconazole group, 90% in the intrastromal amphotericin B group and 95% in the intrastromal natamycin group); albeit, there was noticeably more deep vascularization in the intrastromal amphotericin arm.

**Table 1:** Outcomes of intrastromal injections in the management of mycotic keratitis.

Author	Year	Intervention	Indication	n	Healing Rate	Main Cultures	Mean Healing Duration	Special Comments
Prakash et al. [37]	2008	IS VCZ as an adjuvant to topical NTM and VCZ	Deep recalcitrant fungal keratitis	3	100%	*Fusarium* spp. and *Aspergillus*	18.6 ± 4 days	Targeted delivery of VCZ is safe and effective
Tu [45]	2009	IS VCZ with topical capsofungin or topical fluconazole	Alternaria keratitis not responding to standard therapy	3	100%	*Alternaria*		1 case needed IS VCZ with topical capsofungin
Sharma et al. [40]	2011	IS VCZ after topical NTM, VCZ and oral VCZ	Recalcitrant fungal keratitis	12	83% healed, all of them received >1 injection	*Aspergillus* and *Fusarium*	39.7567.62 days	IS VCZ is effective in recalcitrant fungal keratitis
Sharma et al. [38]	2013	Topical vs. IS VCZ as an adjuvant to topical NTM	Recalcitrant fungal keratitis	40	95% in topical VCZ vs. 80% in IS VCZ (*p* = 0.34)	*Aspergillus* (30%), *Fusarium* (17.5%)	28.9 ± 19.1 days in topical VCZ vs. 36.1 ± 20.2 days in IS VCZ	IS injections with VCZ are not superior to topical VCZ
Tu and Hu et al. [43]	2014	IS VCZ and IS AMB	Late-onset post-DSAEK interface fungal keratitis	2	100%	*Candida* species	3 weeks	IS antifungal therapy is useful in preserving graft viability.
Kalaiselvi et al. [42]	2015	IS VCZ after topical NTM and VCZ	Deep recalcitrant fungal keratitis	25	72%, of which 4 patients needed >1 injection	*Fusarium* (52%), *Aspergillus* (16%)	45.68 ± 11.49 days	IS VCZ is safe and effective in treating deep recalcitrant fungal keratitis
Nada et al. [48]	2017	IS AMB and topical FCZ (group A) vs. topical AMB (group B)	Fungal keratitis	68	- 82.9% in combined therapy - 59.3% in topical AMB	*Candida* and *Aspergillus*	24 ± 6.42 days vs. 39.66 ± 13.6 days	
Konar et al. [41]	2019	IS VCZ after topical NTM and VCZ	Recalcitrant mycotic keratitis	20	70% healed and 15 patients required more than 1 injection (between 2 and 7 injections)	*Fusarium* (30%)	35.5 ± 9.22 days	IS VCZ is proven to be safe in recalcitrant mycotic keratitis
Narayana et al. [39]	2019	IS VCZ + topical NTM vs. topical NTM alone	Primary treatment of severe filamentous fungal ulcers	70	- 77% in IS VCZ and topical NTM - 91% in topical NTM alone	*Fusarium* (27%), *Aspergillus* (24%)		IS VCZ shows no benefit as an adjuvant to NTM in primary treatment
Saluja et al. [55]	2021	IS NTM after 2 weeks of topical NTM	Recalcitrant fungal keratitis	20	95% healed and 30% needed >1 injection	*Aspergillus* (60%) and *Fusarium* (40%)	35.3 ± 6.4 days	IS of novel formulation of NTM plays a promising role in management of recalcitrant fungal keratitis
Saluja et al. [54]	2021	IS VCZ vs. IS AMB vs. IS NTM after two weeks of topical NTM	Recalcitrant fungal keratitis	60	- 95% in IS VCZ group - 90% in IS AMB group - 95% in IS NTM group	*Aspergillus* (53%) and *Fusarium* (40%)	- 34 ± 5.2 days in IS NTM group - 36.1 ± 4.8 days in IS VCZ group - 39.2 ± 7.2 days in IS AMB group	All three antifungal agents show good therapeutic response in recalcitrant fungal keratitis

n, number of cases; IS, intrastromal; VCZ, voriconazole; NTM, natamycin; AMB, amphotericin B; DSAEK, Descemet Stripping Automated Endothelial Keratoplasty.

Additionally, Saluja et al. [59] showed promising results of an intrastromal natamycin composition (10 µm/0.1 mL, sterile water-soluble natamycin complex) as an adjuvant treatment for resistant fungal keratitis to support the use of intrastromal natamycin injection. In a prospective interventional pilot research, twenty eyes with microbiologically confirmed resistant fungal keratitis that did not respond to topical natamycin 5% were treated with water-soluble intrastromal natamycin injections. *Aspergillus* spp. (60%) and *Fusarium* spp. (40%) were the most frequently isolated species. The total cure rate was 95%; the remaining 5% had penetrating keratoplasty because they did not respond to therapy. Epithelial defects, stromal infiltrates, and hypopyon resolution took an average of 34 ± 5.2 days, 35 ± 6.4 days, and 15 ± 2.5 days, respectively. However, deep vascularization repair and cataract development were seen in 31% and 68.42% of cases, respectively [59]. Natamycin is usually administered as a suspension for topical fungal keratitis since it is insoluble in an aqueous solution. It might be converted into a soluble form suited for intrastromal injection by employing the inclusion complexion approach. This procedure most likely extended the distribution of natamycin to the infection site, which eventually led to a more effective microbiological cure. Additionally, the optically transparent and preservative-free intrastromal formulation promoted epithelial defect repair and improved patient monitoring.

Additional research with bigger sample numbers must be conducted in this area for the new natamycin solution to be more thoroughly validated.

### 3.3. Viral Keratitis

In contrast to bacterial and fungal keratitis, viral keratitis has the potential to progress to chronic and recurring stages. In many affluent nations, the herpes simplex virus (HSV) is the most frequent cause of unilateral infectious corneal blindness. Varicella-zoster virus (VZV) keratitis and cytomegalovirus (CMV) keratitis are less frequent types of viral keratitis [60].

Antiviral drugs and adjuvant topical corticosteroids are indications of topical therapy for viral keratitis. In Europe, topical acyclovir is the first-line therapy for HSV keratitis due to its effectiveness and minimal toxicity. A relatively recent synthetic medication with broader antiviral protection is ganciclovir. Topical ganciclovir is beneficial in treating keratitis brought on by CMV in addition to HSV and VZV [61].

According to our knowledge, just one publication has been published on intrastromal antiviral injection in human viral keratitis. A retrospective study of the therapy of nummular keratitis with ganciclovir and depot betamethasone intrastromal injection has been conducted. Out of 21 eyes treated in this way, 18 eyes (85.71%) remained asymptomatic after the therapy was completed. Over the course of a mean follow-up period of 22.90 months, this improvement was maintained. A viable option for the treatment of nummular keratitis is a depot of betamethasone combined with ganciclovir used intravenously, according to this case series study [62].

### 3.4. The Technique of Intrastromal Injection

#### 3.4.1. Preparation of Intrastromal Drug Concentration

##### Amphotericin B

The 50 mg of amphotericin B powder from the vial were reconstituted with 10 mL of 5% dextrose to create intrastromal amphotericin B (5 µg/0.1 mL). To reach a concentration of 0.5 mg in 10 mL (50 µg/mL) from this solution, 0.1 mL was further diluted with 9.9 mL of 5% dextrose; 0.1 mL of this solution contained 5 µg AMB [54].

##### Voriconazole

The dose of intrastromal voriconazole is 50 µg/0.1 mL and is prepared by adding 20 mL Ringer lactate solution in 200 mg powder of voriconazole to obtain 20 mL drug (concentration 10 mg in 1 mL). A total of 1 mL of this solution is diluted further with 19 mL Ringer lactate to result in a concentration of 0.5 mg/mL (50 µg/0.1 mL) [37].

#### 3.4.2. Injection Procedure

Intrastromal injections can be given under topical anesthesia. Patients who are unwilling to cooperate may receive intrastromal injections with peribulbar anesthetic. The preloaded medicine should be delivered under an operating microscope in a completely aseptic circumstance. A sterile drape was applied to the patient after washing the periocular region with 10% povidone iodine and the conjunctival sac area with 5% povidone iodine. Using a 30-gauge or 26-gauge needle, the reconstituted solution is put into a 1 mL tuberculin syringe. The abscess at mid-stromal level, which is the targeted location of drug deposition, is merely reached by inserting the needle obliquely from the unaffected area with the bevel up or down. The quantity of corneal hydration is then utilized as a reference to determine the extent of coverage once the medicine has been administered. The plunger is gently retracted when the appropriate level of hydration has been reached to guarantee capillary column termination and to stop medication back-leakage. To create a drug deposit around the circle of the lesion, five split doses are administered around the abscess.

This is performed so that the abscess along each meridian is covered by a centripetally directed progressive wave of fluid. A barrage of intrastromal medication will build all the way around the abscess due to the circumferential injections. This procedure can be repeated, with at least 72 h between injections [37,38].

#### 3.4.3. Limitations and Complications of the Procedure

Since conducting any intervention via the normal cornea in the presence of keratitis may lead to a new focus of infection, the main drawback of this technique may be the spread of infection. While performing the treatment in a hazy cornea, there is also a possibility of unintentional anterior chamber entrance; however, the perforation may not be entirely attributable to intrastromal injection, and the underlying sterile keratolysis might have contributed to it. In addition, hyphema and intrastromal bleeding have been documented as consequences. Overall, only a very tiny percentage of patients had any procedure complications [40].

In addition, Descemet membrane detachment after intrastromal injection in individuals who had prior lamellar keratoplasty has been documented. According to the authors, an injury to the interface vessels may have caused hemorrhagic Descemet detachment, and Descemet rupture or direct drug injection beneath the Descemet membrane may also have caused Descemet detachment [23].

## 4. Conclusions

The authors claim that intrastromal medication administration was able to directly target the nidus of the infection and led to a prompter resolution, even if the keratitis may have resolved with a longer duration of therapy in certain instances using the usual topical therapy. Further investigation is needed to identify the safest antibiotic option and the minimal effective dose and concentration needed for diverse pathogens intrastromally in bacterial keratitis. In spite of these concerns, intrastromal antibacterial medicines could provide a suitable substitute option for treating severe refractory keratitis. Furthermore, they could be most effective in situations involving deep stromal bacterial infections, infections caused by bacteria that form biofilms, or other situations where it is thought that topical antibiotics could not penetrate well enough to the infection site. In situations of keratitis resistant to topical medication, physicians should give intrastromal antibiotics special consideration before performing penetrating keratoplasty.

Intrastromal injections of voriconazole seem to be the most effective first-line intrastromal antifungal drug, despite conflicting findings about outcomes after treatment of fungal keratitis. Due to variations in microbiological profiles and anticipated results, such as the distinction between clinical resolution and microbiological cure, more clinical investigations are required. As a consequence, determining an organism’s previous susceptibility to the medicine being used to treat the infection may help to improve surgical outcomes. We feel that intrastromal injections may be effective as a safe and useful adjuvant to conventional treatment for the management of persistent infectious keratitis based on the success of the intrastromal agents in the published publications. However, further research in this area is required.

It is necessary to use surgery in situations of refractory keratitis, especially if there is a high risk of perforation. Intrastromal injection of various medications, however, can offer a nonsurgical treatment alternative.

The benefits of employing intrastromal injections have been highlighted, including direct drug delivery to the region that is contaminated while also functioning as a depot to provide a delayed release of the antimicrobial agent, overcoming the difficulty of limited penetration, and reducing epithelial toxicity. To our knowledge, there has not been a significant clinical trial investigation yet on the safety and effectiveness of the intrastromal injection approach. Despite the fact that there have been no reports of this technique having any major safety issues so far, a more in-depth study is mandatory.

As evidenced by the existing literature, intrastromal antifungal injections demonstrate greater efficacy compared to antibacterial injections. This can be attributed to the superior corneal penetration and effectiveness of topical antibacterial agents in managing bacterial keratitis cases. Although intrastromal injections may be employed for bacterial keratitis, the necessity for this technique is not as imperative as for fungal keratitis, given the enhanced performance of topical antibacterial agents.

Based on our study of the literature and the encouraging findings reported in the majority of the articles that have been published, intrastromal antimicrobial drug injection provides a new option for the management of complex and recalcitrant infectious keratitis.

## Data Availability

Not applicable.
